# Surgical Clinic of Prof. Edmund Andrews

**Published:** 1881-07

**Authors:** 


### Clinical Reports.

ARTICLE V.

SURGICAL CLINIC OF PROF. EDMUND ANDREWS.

*Litholapaxy.*

It is doubtful whether Prof. Bigelow is justified in coining this new name for a mere modification of lithotrity. It is best to be very slow to inflict new technical terms upon the memory of students.

The word is compounded from the two Greek terms, *lithos*, a stone, and *lapaxis*, the action of purgative medicines on the bowels. Literally it would mean "stone-purging." The essence of the operation consists in removing the whole stone at one operation, instead of doing it little by little at several sittings. For this purpose Prof. Bigelow has devised a wash bottle and tubes far superior to those of Mr. Clover, and by the great size of his tubes immensely facilitates the evacuation of the fragments. He has also modified the lithotrite, so that it enters the bladder easier, crushes the stone faster, and is less liable to pinch the folds of the bladder, than the old instruments. Nevertheless his operation has hitherto found little favor among Chicago surgeons, but for my part, believing that small stones in adults ought generally to be removed by crushing rather than cutting, I am prepared to look favorably on Bigelow's plan in many cases, for I have had so much experience in the vexations of slow lithotrity, that I rejoice heartily in any improvement that can combine the safety of lithotrity with the promptness of lithotomy. The following cases occurred at the clinic during the past month:

*Case* 11,348.—This patient was admitted in excellent general health, so that his system scarcely required any preparation. Ether having been administered, the large lithotrite of Bigelow was introduced, and the scale on the staff, after the stone was seized, showed it to have the diameter of two and a half centimeters, or about seven-eighths of an inch. In twenty-five minutes the stone was so completely crushed and washed out, that not the smallest particle could be found by a delicate searcher with the auscultating attachment. In order to introduce Bigelow's large tubes it became necessary to incise the meatus, when they entered without difficulty, and their great superiority over the smaller tubes of Mr. Clover was strikingly manifest. The crushed stone seemed to be sucked up and brought out with the sweep of a small maelstrom.

The patient, on awaking from his ether, declared he had not been so comfortable for months, and could hardly be kept in bed the rest of the day. The following morning he got up in spite of remonstrance, declared that he was perfectly cured and wanted to go home. I explained to him that it was necessary to stay and be sounded a few times to make sure of the completeness of the operation. This pacified him for a few hours, but in the afternoon he declared that he *knew* his cure was complete, and that he never felt better in his life; so, suiting the action to the word, he marched to the depot and took the cars for home.

This imprudent course caused no injury, so far as I can learn, but it is not to be recommended to others for imitation. The case, however, showed in a striking manner how much superior rapid lithotrity is in some instances to either slow lithotrity, or to lithotomy.

*Case* 11,367.—This patient entered with a stone which, when grasped by the lithotrite, measured eight millimeters in diameter, or about a quarter of an inch. Nothwithstanding the small size of the calculus, the bladder was more sacculated and irritable than in the former case. The urethra was small, and even after incision of the meatus would not admit Bigelow's large tubes. As the patient could not stay for gradual dilatation, I proceeded by attaching Mr. Clover's tubes to Bigelow's energetic wash bottle. The inferiority of the Clover tubes was manifest, and though the crushing was readily effected, the operation lasted under ether thirty-five minutes. In both patients Bigelow's splendid lithotrite showed its superiority to those of Sir Henry Thompson, and of Sir William Ferguson. It seizes easily, and with the least possible liability of grasping the folds of the bladder between the jaws.

The patient awakened and had no shock or chill. He was tractable, lay in bed a few days as ordered, and, after being resounded to determine the completeness of the operation, went home cured on the sixth day.

TURPENTINE TO PREVENT VOMITING UNDER ETHER.

There has been a statement going the rounds of the profession that a dram of spts. turpentine in each pound of ether will prevent anæsthetic vomiting. Hoping to derive advantage from this plan, I had the ether used at the clinic terebinthinated for a time, and a record of the results kept to compare with those of the non-terebinthinated article. The comparison resulted as follows:

Total number anæsthetized during the experiments, 102; patients taking pure ether, 31; number who vomited, 6; per cent. vomited, 19. Patients taking terebinthinated ether, 71; number who vomited, 21; per cent. vomited, 30.

It appears, therefore, that of those who took pure ether, only nineteen per cent. vomited, while of those who took the turpentine and ether, thirty per cent. met that accident. It is needless to say that the turpentine was ignominiously expelled from the clinic. No. 6, Sixteenth street, Chicago. E. A.

---

STUDY OF CAUSES OF CONSUMPTION.—Dr. Edward Playter, of Toronto, Ontario, has undertaken a systematic study of the causes of consumption. As an aid in this study he requests members of the profession having well-marked cases of consumption now under treatment to send him their address, in order that he may send a list of questions concerning the consumptive patient to which he wishes replies. A study based on a sufficient number of replies must yield valuable results.

